# The Influence of Sex Hormones in Liver Function and Disease

**DOI:** 10.3390/cells12121604

**Published:** 2023-06-11

**Authors:** Alvile Kasarinaite, Matthew Sinton, Philippa T. K. Saunders, David C. Hay

**Affiliations:** 1 Centre for Regenerative Medicine, Institute for Regeneration and Repair, The University of Edinburgh, Edinburgh BioQuarter, Edinburgh EH16 4UU, UK; 2 School of Biodiversity, One Health, and Veterinary Medicine, University of Glasgow, Glasgow G61 1QH, UK; 3 Wellcome Centre for Integrative Parasitology, University of Glasgow, Glasgow G12 9TA, UK; 4 Centre for Inflammation Research, Institute for Regeneration and Repair, The University of Edinburgh, Edinburgh BioQuarter, Edinburgh EH16 4UU, UK

**Keywords:** liver, NAFLD, sex hormones, estrogen, testosterone, HRT, immune response, in vitro models, human PSCs, tissue engineering

## Abstract

The liver performs a multitude of bodily functions, whilst retaining the ability to regenerate damaged tissue. In this review, we discuss sex steroid biology, regulation of mammalian liver physiology and the development of new model systems to improve our understanding of liver biology in health and disease. A major risk factor for the development of liver disease is hepatic fibrosis. Key drivers of this process are metabolic dysfunction and pathologic activation of the immune system. Although non-alcoholic fatty liver disease (NAFLD) is largely regarded as benign, it does progress to non-alcoholic steatohepatitis in a subset of patients, increasing their risk of developing cirrhosis and hepatocellular carcinoma. NAFLD susceptibility varies across the population, with obesity and insulin resistance playing a strong role in the disease development. Additionally, sex and age have been identified as important risk factors. In addition to the regulation of liver biochemistry, sex hormones also regulate the immune system, with sexual dimorphism described for both innate and adaptive immune responses. Therefore, sex differences in liver metabolism, immunity and their interplay are important factors to consider when designing, studying and developing therapeutic strategies to treat human liver disease. The purpose of this review is to provide the reader with a general overview of sex steroid biology and their regulation of mammalian liver physiology.

## 1. Introduction

The liver is a remarkable organ, which coordinates a multitude of critical functions, whilst retaining the ability to dramatically remodel and regenerate damaged tissue [1]. In this review, we explore the role that sex steroids play in maintaining normal liver function and discuss the importance of developing new model systems to improve our understanding of the underpinning biology.

The liver is composed of four lobes which are subdivided into lobule structures. These are hexagonal in appearance, with each corner displaying the portal triad that consists of the portal vein, bile duct and hepatic artery [2]. The major metabolic cell type of the liver is the hepatocyte, which accounts for ~70% of the organ’s mass. Hepatic function is regulated by a number of factors, including oxygen availability, growth factor signaling, extracellular matrix interactions and communication with the non-parenchymal cell compartment [3]. This is key to the liver’s ability to rapidly filter and detoxify waste products, thereby reconditioning the blood [4].

Although the liver is an exceptionally regenerative organ, chronic exposure to toxins can ultimately result in scar tissue formation. This does not only have consequences for organ function but is also a major barrier for liver tissue remodeling and regeneration. If this process continues unchecked, extensive scarring can prevent regeneration, leading to organ failure and death. Currently, orthotopic liver transplantation is the only effective treatment for acute organ failure or end-stage disease. However, due to the limited number of donor organs, as well as complex surgery and complications associated with lifelong immunosuppression, this approach is not a universal treatment option for all patients [5]. Therefore, a number of alternative approaches have been employed to restore basic liver function in humans. Adult donor hepatocytes have been successfully transplanted to treat metabolic liver diseases such as urea cycle defects, Crigler–Najjar syndrome type I and glycogen storage disease type 1a [6]; however, the cell transplants are not a permanent solution as they are eventually cleared by the immune system [7]. Due to these current treatment limitations, tissue engineering and the development of new sources of liver tissue, such as adult progenitors and pluripotent stem cell-derived liver tissue, represent attractive renewable systems of the future to treat failing liver function in humans.

Liver scarring occurs as a result of imbalanced matrix deposition and remodeling, resulting in the buildup of fibrotic tissue [8]. This is a complex process that depends on the interaction of both resident liver cells as well as recruited immune cells. Central to liver fibrosis is the activation of hepatic stellate cells (HSC) by the immune system. Key factors involved in HSC activation include the pro-inflammatory and pro-fibrogenic cytokines, TGF-α and TGF-β, which increase cell proliferation and promote a myofibroblast phenotype. In parallel, anti-fibrogenic cytokines (including IL-10, IFN-α, IFN-γ) are also released from immune cells to moderate the pro-fibrogenic response. The balance of these key players results in a net pro- or anti-fibrogenic HSC response (for a review see Acharya et al., 2021 [9]). It is important to note that liver fibrosis is reversible in the early stages of disease [10]. The major events promoting recovery are myofibroblast apoptosis and macrophage transition from a pro-inflammatory to a pro-resolution phenotype [11]. During these processes, deposited ECM is remodeled via matrix metalloproteinases, specifically acting on collagen, gelatin and elastin [12].

A major risk factor for the development of liver disease is obesity, which is associated with elevated blood glucose, cholesterol and triglyceride levels, as well as excess adipose deposition around the waist. Together, these factors are termed metabolic syndrome (MetS), which underpins non-alcoholic fatty liver disease (NAFLD) [13]. Specifically, NAFLD is defined by the development of macrovesicular steatosis, whereby hepatocytes accumulate triglycerides. Although steatosis is largely regarded as benign, it does progress to non-alcoholic steatohepatitis (NASH) in approximately 30% of patients, increasing their risk of developing fibrosis, cirrhosis and hepatocellular carcinoma in the future [13]. To date, there are no specific therapeutics available to reverse or treat NAFLD or NASH. The only effective intervention for obesity-induced disease is through weight reduction following invasive and permanent bariatric surgery [14], highlighting a clinical unmet need to find alternative less invasive treatment strategies. NAFLD susceptibility varies across the population, with obesity and insulin resistance playing a strong role in the disease process. Sex hormones, notably estrogens and androgens, also contribute to the risk of developing liver disease. Specifically, epidemiological studies have reported that genetic sex and age are important risk factors for NAFLD. NAFLD is twice as common in postmenopausal women as in premenopausal women consistent with a protective role for estrogens, but the mechanisms responsible remain under-explored [15]. In addition to the regulation of biochemical processes within the liver, sex hormones also regulate the immune system (for a review see Taneja et al., 2018 [16]), and sexual dimorphism has been described for innate and adaptive immune responses [17]. These are important factors to consider when designing therapeutic strategies for hepatic disease, which may need to be stratified for men or women and take into account therapies such as hormone replacement therapy (HRT) in postmenopausal women and in exogenous hormones in transgender people.

To model the relationship between healthy and diseased liver, as well as sex hormone and immune interplay, we suggest implementing in vitro models. Although there are a number of in vitro approaches to study liver disease, they do possess drawbacks. For example, cell line-based models display perturbed genetic and metabolic function, arising from malignant transformation and/or immortalization. Whereas primary mouse and human hepatocyte-based systems display species differences and unstable phenotype post isolation, limiting their application. Additionally, human hepatocytes are commonly isolated from transplant-rejected livers, often fatty in nature, which may adversely affect their performance [18]. Therefore, we and others have opted to use pluripotent stem cell- derived systems as a renewable source of human tissue to model liver disease (for a review see Szkolnicka et al., 2016 [19]).

Given the international prevalence of NAFLD and the complexity of disease progression, we decided to write this review article, providing insight into the role of hormonal signaling in mammalian liver physiology.

## 2. Sex Steroid Biosynthesis, Signaling and Regulation of Liver Function

Sex steroid hormones are synthesized by the gonads and adrenals, and to a lesser extent in liver and adipose tissue [20,21]. The most potent sex steroids are estrogens, androgens and progestins, all of which instruct cell and tissue function and may also contribute to development of pathologies including malignant transformation [22]. To date, most attention has been paid to the impact of sex steroids on reproductive tissues [23,24] and hormone-dependent cancers [25,26]. In this review, we focus on estrogen and androgen signaling, and their impact on liver biology.

### 2.1. Overview of Sex Hormone Biosynthesis

**Figure 1:**
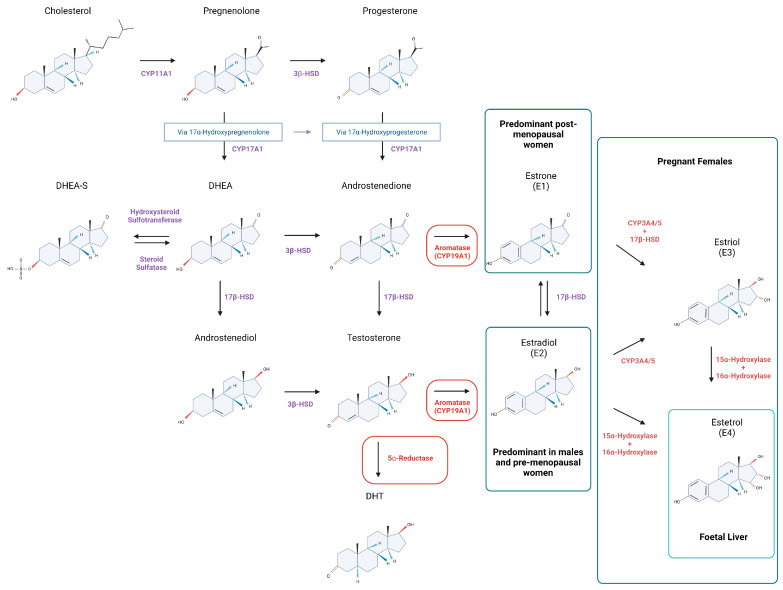
Sex hormone biosynthesis in vivo. Overview of testosterone and estrogen biosynthesis in humans. Cholesterol is processed into different DHEA and progesterone via several steps, leading to testosterone synthesis via androstenediol by 3β-HSD or androstenedione by 17β-HSD. Testosterone can be further metabolized to its potent form, DHT, by 5α-reductase. E2, can be synthesized from testosterone by aromatase or via E1 by 17β-HSD type 1. The other two forms of estrogen, E3 and E4, are detected within pregnant women and the fetal liver. CYP11A1, cytochrome P450 cholesterol side-chain cleavage enzyme; 3β-HSD, 3β-hydroxysteroid dehydrogenase D5-D4 isomerase; DHEA, dehydroepiandrosterone; DHEA-S, dehydroepiandrosterone sulphate; CYP17A1, cytochrome P450 17α-hydroxylase; 17β-HSD, 17β-hydroxysteroid dehydrogenase; DHT, dihydrotestosterone; CYP19A1, cytochrome P450 aromatase; E1, estrone; E2, estradiol; E3, estriol; E4, estetrol; CYP3A4/5, cytochrome P450 3A4/5.

Cholesterol translocation to the mitochondrial membrane is the first step of sex hormone biosynthesis within a cell [27,28] (Figure 1). Thereafter, cholesterol is converted to pregnenolone by CYP11A1, a member of the cytochrome P450 superfamily of enzymes [29,30] at the start of a multistep enzymatic cascade that results in generation of estrogens and androgens (Figure 1). Specifically, pregnenolone is converted to progesterone by 3β-Hydroxysteroid dehydrogenase (3β-HSD). In addition, 3β-HSD may also metabolize DHEA (formed from pregnenolone via 17α-hydroxyprogesterone) to androstenedione or androstenediol to testosterone, T (metabolized from DHEA by 17β-Hydroxysteroid dehydrogenase, 17β-HSD) [27,29,30,31]. Aromatase (CYP19A1) can catalyze the formation of E1 (estrone) [27,29,30,31] or E2 (estradiol) from androstenedione and T, respectively [27,29,31]. As well as acting as a precursor for E2, T may also be processed to its potent form dihydrotestosterone (DHT) by 5α-reductase [29,30,31], something that predominantly happens within peripheral tissues such as prostate [32]. Intraconversion of E1 and E2 is regulated with 17β-HSDs of which there are ~14 isoforms in humans [27,29] (Figure 1). 17β-HSD isoforms 2, 4, 5 (AKR1C3), 6–7 and 10–14 are all expressed in the liver [33,34,35,36,37]. In the liver, low aromatase levels in hepatocytes have been associated with disorders such as cirrhosis and steatosis in men [38]. Studies in male mice have shown that targeted deletion of the aromatase gene (ArKO) resulted in glucose and insulin intolerance [39] (Table 1). Whilst in female mice, aromatase deficiency has been associated with elevated T levels, that in turn lead to impaired liver metabolism [40] (Table 1). Other enzyme deficiencies may also contribute to liver disease; one example being 5α-reductase type 1 (Srd5a1) knockout which leads to steatosis and fibrosis in male mice [41,42] (Table 1).

### 2.2. Circulating Hormones in Women

Estrogens are a group of female sex hormones, composed of E1, E2, estriol (E3) and estetrol (E4, fetal liver) [27,29,43,44] (Figure 1). All four estrogens can bind to nuclear and membrane estrogen receptors, however, they have different binding affinities and effects on downstream gene expression. E2 is considered a ‘strong’ estrogen, with high affinity for estrogen receptors, whilst E1 and E3 are deemed to be ‘weak’ estrogens based on analysis of receptor-mediated signaling in cancer cells [45]. The relative abundance of circulating estrogen depends on a women’s reproductive status. For example, E3 and E4 are predominantly detected during pregnancy, with E4 exclusively converted from E2 and E3 by fetal liver enzymes [43,44] (Figure 1). E2 is the predominant form of circulating estrogen in premenopausal women [46], whilst E1 concentrations increase during menopause [29,47]. In premenopausal women, E2 is produced by granulosa cells in ovarian follicles, whilst after menopause cessation of ovulation means the hormone is synthesized in extragonadal tissues [48]. Normal levels of total E2 in premenopausal women range between 30 and 400 pg/mL (depending on ovarian function), but this drops to 0–30 pg/mL in postmenopausal women [49].

Women also produce testosterone, which is synthesized in the adrenal gland, ovary and extragonadal tissues, including the liver [50]. Normal measurements range from 0.5 to 2.4 nmol/L [51]. During menopause, there is a relative increase in androgen levels associated with reduced conversion to estrogens in the ovaries [52]. Some reproductive disorders such as polycystic ovarian syndrome (PCOS) are associated with excess circulating androgens and the development of metabolic syndrome [53]. PCOS is often also associated with obesity and is an example of the impact of disruption in androgen:estrogen balance in women with implications for impaired lipid and glucose metabolism in the liver [54,55]. Excess androgens in women with PCOS also place them at higher risk of NAFLD with a recent review highlighting the importance of SHBG, a steroid-binding protein produced in the liver in the regulation of bioavailable androgens in women with a suggestion that levels of SHBG could be used as a biomarker for NAFLD [56].

### 2.3. Circulating Hormones in Men

Androgens are considered the primary male sex hormones. There are several forms of circulating androgens, with T and DHT acting as potent ligands for androgen receptors (AR) [57]. Normal levels of overall T in men are 10–35 nmol/L [51]. Free testosterone and testosterone loosely bound to albumin in blood can interact with AR, but the tight complex of testosterone bound to SHBG cannot bind to AR in target tissues [58]. Notably, circulating levels of SHBG are influenced by liver function and by obesity with implications of androgen action in tissues such as muscle [59,60]. DHT has a higher affinity for AR in binding studies and is considered a more potent receptor activator than testosterone [61]. In contrast to testosterone, only 20% of DHT is secreted by testes in men, whilst the rest is converted by 5α-reductase in extragonadal tissues [62,63]. Both androgen and AR levels decrease in men with age [64,65,66].

In contrast to women, E2 production in men does not primarily rely on the gonadal tissues, but rather depends on the aromatization of testosterone in both gonads and extragonadal tissues, such as adipose tissue [67]. Normal circulating E2 levels are between 10 and 50 pg/mL [49]. Decreased SHBG in obese men can increase the amount of T available for conversion to E2 and the increase in fat mass results in upregulated aromatase activity and consequently higher production of E2 [60,68]. With relevance to the liver, the decrease of testosterone levels and the increase of circulating estrogen is associated with visceral adiposity, insulin resistance and MetS [58,63,69], all of which are detrimental to health.

### 2.4. Receptor-Dependent Signaling by Estrogens and Androgens

**Figure 2:**
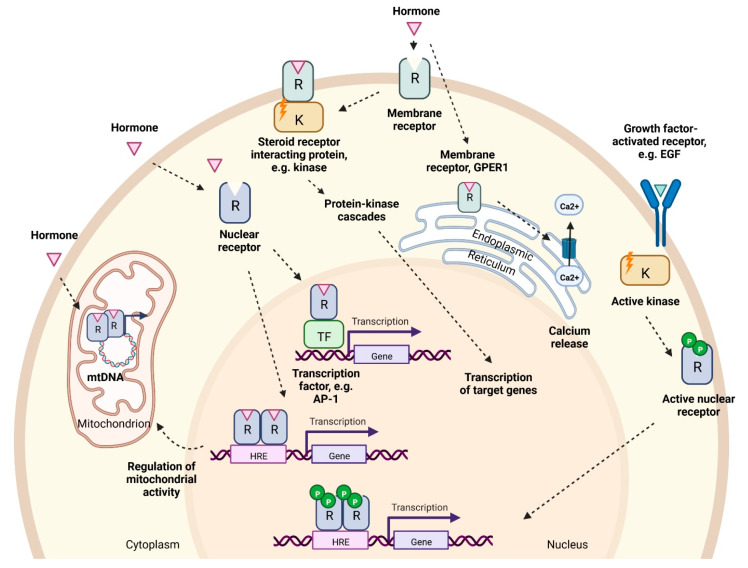
Genomic and non-genomic effects of hormonal signaling. Several hormone pathways exist. Ligand-bound nuclear hormone receptors (R) can either directly activate hormone response elements (HREs), located on the regulatory sequences of target genes, or they bind to other transcription factors (e.g., AP-1). Intracellular nuclear receptors can also be activated in the absence of hormone via growth factors (e.g., EGF) via kinase-mediated phosphorylation (ligand-independent pathway). In the presence of hormone, receptors may also regulate mtDNA transcription. Hormone-activated membrane receptors activate protein-kinase cascades (e.g., MAPK signaling pathway) and regulate the levels of secondary messengers within the cell (e.g., Ca^2+^ intracellular influx via GPER1). R, hormone receptors (nuclear—blue, plasma membrane—green); HRE, hormone response elements; TF, transcription factor; K, kinase; P, phosphorylation.

Ligand binding to estrogen and androgen receptors can induce changes in gene expression and cell function by two main mechanisms broadly referred to as genomic or non-genomic (Figure 2).

### 2.5. Genomic Pathway

Two estrogen receptor genes have been identified in humans encoding alpha and beta receptor subtypes, ERα (ESR1) and ERβ (ESR2). They share >95% amino acid homology in their DNA-binding domains and 59% sequence identity in their ligand-binding domains [70,71]. When the ligand interacts with ERα or ERβ in the target cell, the receptors undergo a conformational change, forming homo- or heterodimers and become associated with other proteins. Thereafter they bind to the estrogen response elements (ERE) in the regulatory regions of target genes, promoting their expression [57]. Alternative gene activation may occur via so called ‘tethered’ mechanisms involving other transcription factors such as AP-1 and Sp-1 that do not require ERE sequences for binding to the promoter regions [72] (Figure 2). In relation to liver, ERα knockout (ERKO) mice display increased insulin resistance and lipid storage in both males and females, leading to NAFLD [73,74] (Table 1).

Androgen binding (T or DHT) to receptors within the cell results in a conformational change in receptor protein, formation of homodimers and binding to androgen response elements (ARE) in target genes [75], or to other transcription factors such as AP-1 [57] (Figure 2). Androgens play a key role in the development and maintenance of male phenotype in men and mice, with ARKO mice having smaller testes, and female-like external genitalia. Specific to the liver, tissue-specific ablation of AR in male mice led to steatosis due to increased de novo lipid synthesis and decreased fatty acid β-oxidation in hepatocytes [76] (Table 1).

### 2.6. Non-Genomic Pathway

The non-genomic activities of sex hormones are associated with rapid activation of downstream targets that are significantly faster than classical ligand-activated receptor pathways [77] (Figure 2). [78]. Estrogens may act via membrane-bound variants of ERα and ERβ, and on G-protein-coupled estrogen receptor (GPER1) [57,79,80]. Estrogen receptors are present on the plasma membrane of every cell, however, their abundance varies depending on the cell type. Both ER variants are expressed within human and rat liver cells, although ERα is known to be expressed at higher levels than ERβ [81]. Estrogen- stimulated ERα and ERβ receptors are also found on mitochondria and are believed to coordinate organelle biology within the cell [82,83]. GPER1 is also present on endoplasmic reticulum, mediating cell apoptosis via Ca^2+^ cascade [84]. Specific to liver, GPER1-KO female mice exhibit higher risk of liver injury [85], whilst GPER1-KO affects aged male mice resulting in fat accumulation and elevated triglyceride levels [85,86] (Table 1).

Androgen receptors present on the plasma membrane also induce a rapid response to testosterone and DHT [87] (Figure 2). The non-genomic activity of androgens involves the production of secondary messengers and the activation of kinase-mediated pathways [87]. Furthermore, AR can also cooperate with ERα/ERβ to promote the activity of c-Src kinase [88], suggesting that E2 and androgen signaling are interconnected. Plasma membrane G protein-coupled receptors are also found to induce intracellular levels of Ca^2+^ upon testosterone binding [87]. AR are also found in mitochondria and regulate OXPHOS and mtDNA homeostasis [89].

### 2.7. Ligand-Independent Signaling Pathway by Estrogens and Androgens

Estrogen receptors can be activated in the absence of estrogen. The phosphorylation of the specific residues of the receptors cause their translocation to the nucleus and the initiation of target gene transcription as described in the genomic pathway section [78]. Estrogen-independent ER activators include epidermal growth factor (EGF), insulin and others [78] (Figure 2).

Androgen-independent mechanisms involve constitutively active AR splice variants, mutations within AR or activation of AR by growth factors [90]. Insulin-like growth factor, EGF and others mediate AR-induced cell cycle regulation and apoptosis within cells [91] (Figure 2).

### 2.8. Estrogen Signaling in Liver

Local estrogen bioavailability can be regulated by liver enzymes which include aromatase and 17β-HSD family members [92,93]. Amongst estrogen receptor subtypes, ERα is reported to be the most abundant ER in both female and male hepatocytes [81,94]. In both sexes, estrogen signaling regulates lipogenesis, glucose and cholesterol homeostasis. Both male and female mice with ERα or liver-specific ERα knockout (ERKO and LERKO, respectively) develop fatty liver consistent with a role for ER-dependent gene expression in liver homeostasis [73,74,95] (Table 1). EREs are found in numerous promoters which exhibit a sex bias in human and rat liver gene expression as has been observed for CYP450 superfamily members, including CYPs 1A2, 3A4 and 4A11 [96,97]. Sex-biased secretion of hepatokines, such as adropin, whose mRNA levels are implicated in development of fatty liver and insulin resistance, also showed estrogen-dependent regulation via ERα in mice [98].

**Figure 3:**
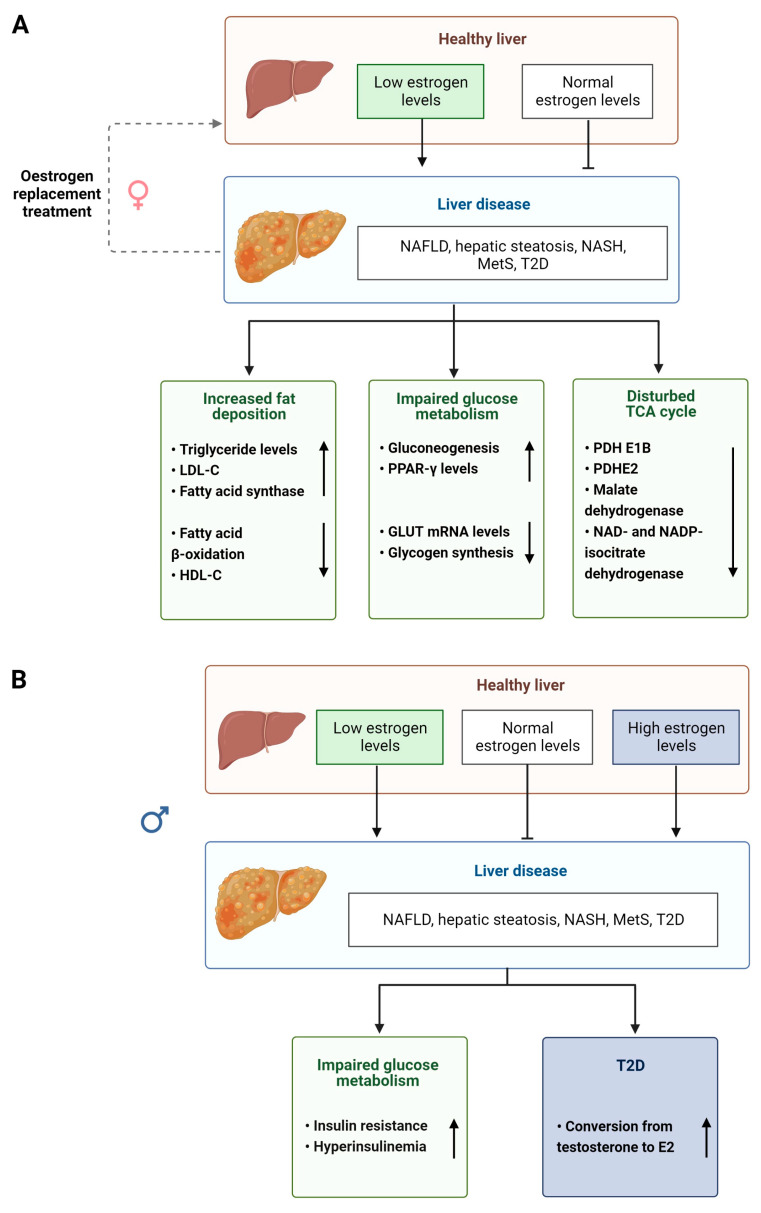
Estradiol effects on liver. (**A**) Low levels of estrogen are associated with liver pathology in females via increased fat storage, impaired glucose and TCA cycle pathways; (**B**) Impaired estrogen signaling is associated with liver pathology in males. Low estrogen promotes liver disease via impaired glucose metabolism. High levels of estrogen promote NAFLD development via T2D. NAFLD, non-alcoholic fatty liver disease; NASH, non-alcoholic steatohepatitis; MetS, metabolic syndrome; T2D, type 2 diabetes; LDL-C, circulating low-density lipoprotein; HDL-C, circulating high-density lipoprotein; PPAR-γ, peroxisome proliferator-activated receptor gamma; GLUT, glucose transporters; PDH E1B, pyruvate dehydrogenase E1 beta; PDHE2, pyruvate dehydrogenase complex subunit E2; E2, estradiol.

The level of bioavailable estrogen is therefore important for normal physiological functioning of the liver and disruption of this signaling axis can have profound effects in both men and women (summarized Figure 3A,B). For example, lower levels of circulating estrogens found in postmenopausal women and in men are associated with an increase in the levels of plasma cholesterol and low-density lipoprotein (LDL) promoting fat accumulation and altering lipid homeostasis in the liver [99,100,101]. It has been shown that GPER1 is more important in aging males [85,86], whereas plasma ERα is more important in female lipid regulation [102,103] (Table 1). The estrogen pathway also plays a role in liver glucose metabolism and homeostasis [104] (Table 1), regulating insulin release, expression of the glucose transporter (GLUT) gene and glycogen synthesis [104,105,106]. It is interesting to note that type 2 diabetes (T2D) is more prevalent in men than in women due to impaired E2 conversion in male livers [107]. Supporting this notion, aromatase-deficient men, but not women, display obesity, insulin resistance and hyperinsulinemia [108]. After menopause, lower levels of circulating estrogens can lead to women developing T2D, MetS [109] and NAFLD. The progression to chronic liver disease, such as NASH and HCC, has also been associated with altered estrogen signaling [110], with HCC being 4 times more common in males than in females until menopause [111].

### 2.9. Androgen Signaling in Liver Pathology

Androgens also regulate metabolism in the liver and perturbations in their biotransformation and signaling can lead to the development of disease [57] (Figure 3). Elderly men with MetS and obesity display significantly reduced serum testosterone levels and increased E2 levels [112,113] (Table 1). However, lipid metabolism can be partially restored in male mice with non-functional ARs and by treatment with T suggesting that androgens have an impact on lipid homeostasis that is independent of functional ARs [114,115] (Table 1). In women, increased androgen levels, such as in those with PCOS (see above) pose an increased risk of visceral adiposity and fatty liver disease [116,117,118].

**Figure 4:**
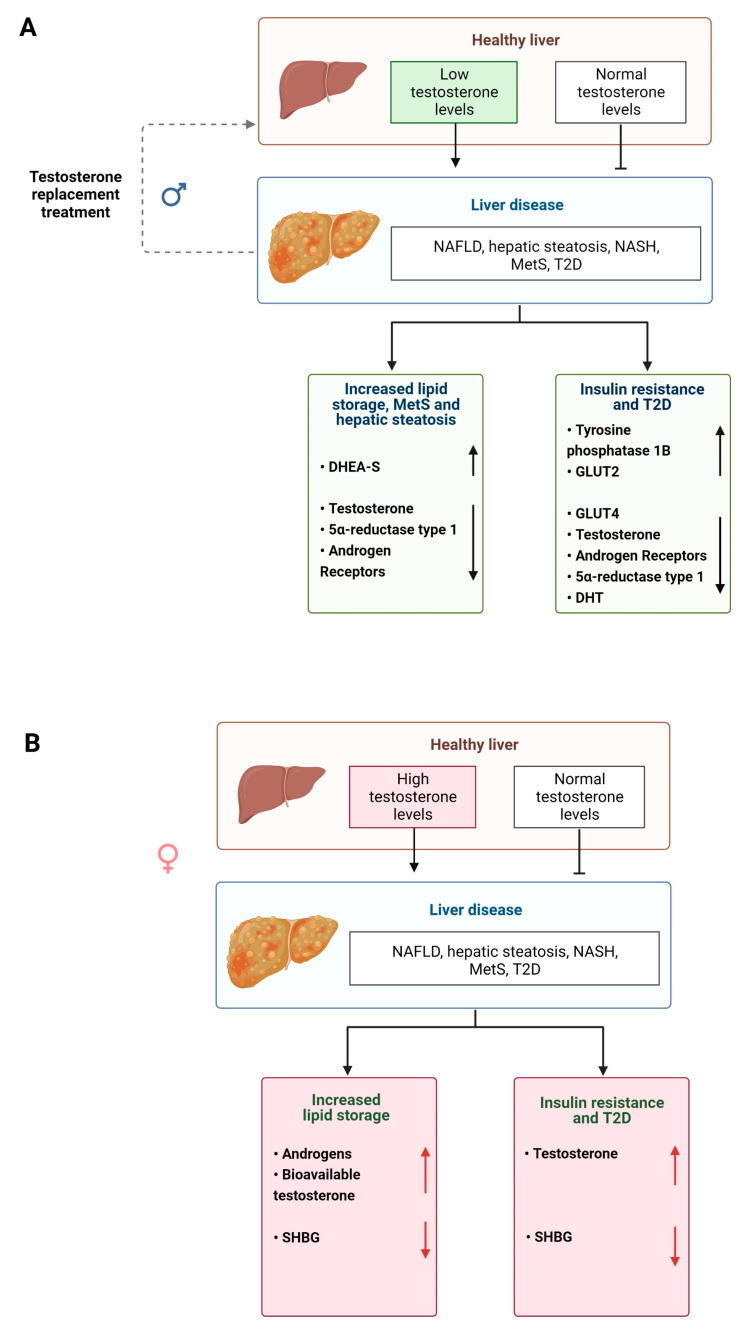
Testosterone effects on liver. (**A**) Low levels of testosterone are associated with liver pathology in males via increased lipid storage, MetS and hepatic steatosis, impaired insulin signaling and T2D; (**B**) High testosterone levels are associated with liver pathology in females via increased lipid storage, impaired glucose metabolism and T2D. NAFLD, non-alcoholic fatty liver disease; NASH, non-alcoholic steatohepatitis; MetS, metabolic syndrome; T2D, type 2 diabetes; DHEA-S, dehydroepiandrosterone sulphate; GLUT2 and 4, glucose transporters 2 and 4; SHBG, sex hormone-binding globulin.

Androgens also act as regulators of glucose metabolism, although differently in men and women (Figure 4A,B). High testosterone levels are associated with decreased risk of insulin resistance and T2D in men but increase the risk in women [119]. In male mice, testosterone and DHT maintain glucose homeostasis. Whereas in castrated male rats, glucose is exported out of the liver, increasing blood glucose levels, which is a risk factor for developing disease [41,120] (Table 1). Reduced DHT levels are also associated with weight gain, hyperinsulinemia, hepatic steatosis and liver fibrosis in male mice [41] (Table 1). When male and female obese and AR knockout mice are compared experimentally, only male mice display decreased insulin sensitivity and elevated levels of protein tyrosine phosphatase 1B [76] (Table 1).

**Table 1:** Summary of the impact of disruptions in synthesis of sex steroids or their receptors in male and female mice and rats.

Animal	Sex	Modification	Phenotype	Reference
Mouse	Male	ArKO (aromatase deficient) in liver and muscle	Obese Hyperglycemia Insulin resistanceUpon E2 administration: recovery to WT phenotype	[39]
Mouse	Female	ArKO	10-fold elevated testosterone compared to wild type (WT)	[40]
Mouse	Male	5α-reductase1-KO (Srd5a1^−^/^−^)	Impaired testosterone to DHT conversion Obesity Hyperinsulinemia Hepatic steatosis Predisposition to hepatic fibrosis	[41,42]
Mouse	Female	MOER (only plasma membrane ERα)	Normal response to E2 in ERK and PI3K activation Abnormal reproductive tract, mammary gland, hormone secretion Obesity without functional nuclear ERα	[102,103]
Mouse	Female	ERKO (ERα knockout) and LERKO (liver-specific ERKO)	Higher body weight than WT Insulin resistance Higher leptin levels Fasting hyperinsulinaemia Hyperglycemia Altered hepatokine production	[73,74,95,98,104]
Mouse	Male	ERKO (ERα knockout)	Similar body weight to WT Fasting hyperinsulinemia Hyperglycemia	[73,74,104]
Mouse	Female	BERKO (ERβ knockout)	Normal body weight	[73]
Mouse	Female	GPER1-KO (Gpr30-LacZ) and high-fat diet supplementation	Young mice: Lower levels of HDL, higher risk of liver injury	[85]
Mouse	Male	GPER1-KO (Gpr30-LacZ) and high-fat diet supplementation	Young mice: No lipid profile changes, reduced expression of liver damage markers (ALAT, ASAT) Old mice: Weight gain, elevated triglyceride levels and cholesterol	[85,86]
Mouse	Male	ARKO (androgen receptor knockout)	Failure to develop male phenotype Reduced testes and serum testosterone levels Decreased fatty acid β-oxidation and PPARα expression Triglyceride accumulation Hepatic steatosis Insulin resistance Leptin resistance Risk of T2D	[76,115,121]
Mouse	Female	ARKO	Impaired ductal system within mammary glands Insulin resistance Hepatic steatosis	[121,122]
Rat Mouse	Female	OVX (ovariectomized)	Removes ovarian E2 Impaired glycogen synthesis Impaired TCA cycle Risk of MetS and NAFLD Altered hepatokine production	[106,123] [98]
Rat Mouse	Male	Castrated Testicular feminized	Disrupted testosterone production Impaired regulation of glucose transporters Elevated blood glucose levels Risk of T2D Increased hepatic lipid deposition	[120] [114]

### 2.10. Hormone Replacement Therapies for NAFLD

Hormonal regulation of liver metabolism is a potential therapeutic target for treatment of human liver disease (Figure 3A and Figure 4A) and has been investigated in clinical trials. For example, when postmenopausal women with T2D received either E2 with or placebo for 6 months, the group which received the active hormone had lower levels of circulating alanine aminotransferase and GGT suggesting decreased fat content within liver [124]. Additionally, a larger study of postmenopausal women demonstrated that NAFLD was reduced in a group undergoing HRT therapy. After menopause, the rise in T may increase the risk in developing NAFLD, suggesting testosterone antagonists could be used. Notably, short-course trials with spironolactone, a competitive inhibitor of AR, showed decreased serum fatty acids and visceral adiposity in women [54].

Similarly, androgen HRT in men reduces liver steatosis. Men with T2D and lowered serum testosterone levels that received testosterone therapy had lower liver fat than compared to the placebo group, as well as decreased liver damage markers [125]. In the same study, men who received testosterone treatment displayed a reduction in absolute liver fat [125].

Although HRT improves liver physiology and function in patients, it also carries risks. Estrogen-only therapy increases a risk for endometrial cancer in menopausal women with a uterus. When considering HRT in women, it is important to compare risks of E-only and combined regimes that may include androgens [126]. Some evidence suggests HRT may be associated with increased risk of breast cancer, dementia, blood clots and stroke with long-term use [127]. Testosterone therapy in men is also not risk-free. The administration of testosterone can accelerate the development of benign prostatic hyperplasia and prostate cancer and increases the risk of breast cancer and cardiovascular disease [128]. Therefore, whilst liver function may be enhanced, improved formulations and better balanced regimes need to be developed to balance out benefit versus risk, particularly with longer term use, for example, amongst hypogonadal men, women with premature menopause and transgender individuals [129].

## 3. The Role of the Immune System in Liver Biology and Metabolism

**Figure 5:**
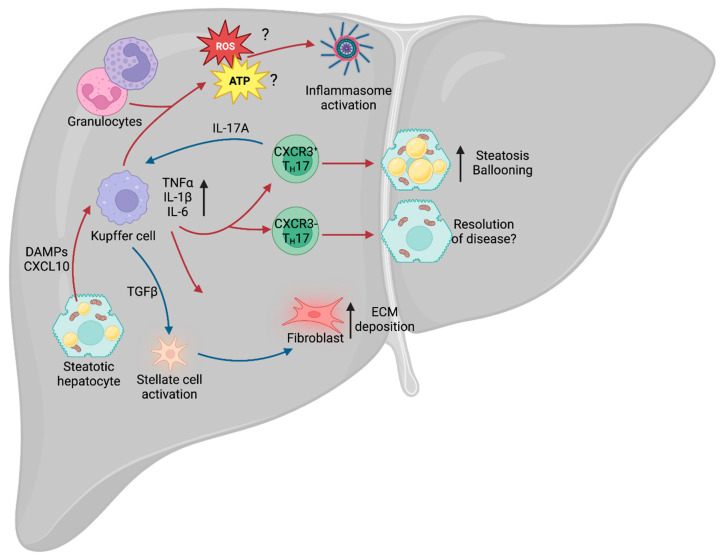
Liver immunity. Schematic of the broad immune changes occurring in the liver during NAFLD. Hepatic steatosis causes the release of DAMPs and CXCL10, leading to activation of the liver-resident macrophages (Kupffer cells). Kupffer cells, in turn, recruit the adaptive arm of the immune system, including T_H_17 cells, which can lead to exacerbation of NAFLD. Additionally, IL-17A stimulates secretion of TGFβ by Kupffer cells, which drives stellate cell activation and release of ECM from fibroblasts. Kupffer cell-derived ROS and ATP also contribute to NAFLD- and NASH-associated inflammation. NAFLD, non-alcoholic fatty liver disease; DAMPs, damage-associated molecular patterns; T_H_17, T helper 17 cell; IL-17A, TH17-derived interleukin 17A; ECM, extracellular matrix; ROS, reactive oxygen species; NASH, non-alcoholic steatohepatitis.

In its healthy state, the liver functions as an immune sentinel, sampling the blood that enters it via the hepatic portal vein, before it reaches the spleen or lymph nodes. This blood is rich in nutrients, as well as any pathogen-derived molecules that are in circulation, such as lipopolysaccharide (LPS). Since the liver filters all of the blood, it is in a prime position to detect these molecules and sound the alarm to the immune system. It is, therefore, unsurprising that damage to the liver leads to a robust immune response, by both the innate and adaptive arms of the immune system (Figure 5). NAFLD leads to lipotoxicity within the liver, which, in turn, causes hepatocytes to become stressed or die, and this process generates inflammatory factors called damage-associated molecular patterns (DAMPs) [130], which are able to trigger activation of the immune system [131]. Whilst the precise mechanisms leading to the progression from NAFLD to NASH remain unclear, there is evidence to suggest that these are, in part, immunological in nature.

### 3.1. Immunity and NAFLD/NASH

Approximately 30% of patients with NAFLD develop an inflammatory phenotype and progress to NASH, with subsequent tissue injury and the development of hepatic fibrosis. However, this key step between the relatively benign NAFLD phenotype and inflammatory NASH remains subject to extensive debate. Chronic metabolic inflammation (metaflammation) is initially promoted during metabolic diseases such as obesity and type 2 diabetes (T2D), and there is evidence to suggest that this fuels the NAFLD–NASH transition. In particular, the adipose tissue has been identified as a major source of inflammatory cytokines, including pro-inflammatory tumor necrosis factor (TNF), interleukin-1β (IL-1β) and IL-6 [132]. In mouse models of hepatic steatosis, systemic deletion of *Tnf* and chemical inhibition of TNF-Receptor-1 reduces the prevalence of steatosis and hepatocellular injury [133,134]. At the hepatic level, lipotoxicity within hepatocytes drives the release of CXCL10 (C-X-C motif ligand 10) [135], which is a chemoattractant, or CXCR3 (C-X-C motif chemokine receptor 3)-expressing Kupffer cells [136], the tissue-resident macrophages of the liver. Upon localization to the site of injury and stimulation of toll-like receptor 4 (TLR4), Kupffer cells also release TNF, IL-1β and IL-6 [137]. TNF-alpha (TNFα) is a major contributor to the inflammatory response, regulating various aspects of sickness behavior during infection, including fever and cachexia. In the context of NAFLD, TNFα was shown to drive an increase in the expression of the genes *Acaca* (acetyl-CoA carboxylase alpha) and *Scd1* (stearoyl-CoA desaturase 1) [133]. *Acaca* encodes a rate-limiting enzyme for fatty acid synthesis [138], whilst *Scd1* encodes a lipogenic enzyme that catalyzes the synthesis of monounsaturated fatty acids [139]. Furthermore, inhibition of the TNFα receptor, TNFR1, improves liver steatosis and insulin resistance [134]. Given that TNFα is a major driver of cachexia during infection, it is paradoxical that it also drives increased expression of these two lipid storage enzymes in NAFLD, suggesting that the role of this cytokine is tissue- and context-dependent.

IL-6 is an important driver of adaptive immune recruitment, selectively controlling T cell recruitment by mediating chemokine secretion [140]. Further to recruitment of T cells, IL-6 also drives polarization of CD4^+^ T cells, inhibiting T helper 1 (T_H_1) and promoting T_H_2 and T_H_17 differentiation [141,142]. In both NAFLD and NASH, patients exhibit increased circulating IFNγ-producing T_H_1 cells, and patients with NASH could be stratified from those with NAFLD by the increase in circulating T_H_17 cells [143]. T_H_17 cells are CD4^+^ cells that express the transcription factor RORγt (retinoid orphan receptor gamma t) and RORα, and are characterized by secretion of IL-17A and IL-17F. It was recently demonstrated that IL-17A and IL-17F are drivers of adipocyte lipid usage in adipocytes during infection and, moreover, promote infection-induced cachexia [144], suggesting that it may drive lipid usage in other cell types, including hepatocytes. As with TNFα, this appears paradoxical, as NAFLD is associated with increased hepatic lipid storage. However, increased expression of TNFα and IL-17A may represent a mechanism by which the liver attempts to mobilize and dispose of excess lipids to restore homeostatic function.

T_H_17 cells are known to expand in the liver of obese humans and mice [145], and multiple rodent models of NAFLD show increased IL-17A signaling through the IL-17A receptor (IL-17RA) [146,147]. The development of NAFLD leads to increased infiltration of non-conventional CXCR3^+^ T_H_17 cells, which can co-express IFNγ. Adoptive transfer of CXCR3^+^ T_H_17 cells into mice with experimental NAFLD increased hepatic damage compared with those given CXCR3^−^ T_H_17 cells [148]. Furthermore, the livers of mice given CXCR3^+^ T_H_17 cells displayed increased triglyceride accumulation and hepatocyte ballooning. Moreover, the presence of CXCR3^+^ T_H_17 cells correlated with increased disease severity in humans, suggesting that this is an evolutionarily conserved aspect of NAFLD and NASH. However, the mechanisms by which these CXCR3^+^ T_H_17 cells exacerbate disease remain unclear. Typically, T_H_17 cell-secreted IL-17A recruits and activates neutrophils [149], a cell type abundant in the liver of NASH patients, but it remains to be seen whether CXCR3^+^ T_H_17 cells exert their effects through neutrophil activation or an as yet unknown mechanism. Further to its role in recruitment of neutrophils, IL-17A signals through IL-17RA on Kupffer cells to promote production of TGF-β1 (transforming growth factor β1) [150], which, in turn, promotes hepatic stellate cell (HSC) activation and extracellular matrix secretion, contributing to NAFLD progression [151].

### 3.2. Immunometabolism in NAFLD/NASH

An emerging area of interest in NAFLD and NASH is immunometabolism. All cells rely on nutrients to function and immune cells are no exception. Indeed, during infection and injury, the immune system requires significant amounts of energy to fuel itself, with different cell types relying on specific nutrients for optimal function. Activation of Kupffer cells leads to enhanced glucose utilization [152] and a rapid increase in aerobic glycolysis (the “Warburg Effect”), whereby cells preferentially rely on glycolysis despite the presence of oxygen [153]. Although glycolysis is less energetically favorable than oxidative phosphorylation (OXPHOS) [154], it carries a number of advantages for immune cells. Early branching of the glycolytic pathway generates precursors for the pentose phosphate pathway (PPP) and de novo nucleotide synthesis, which are required for the function of multiple immune cell types [155]. For example, in macrophages, such as Kupffer cells, the PPP is required to sustain superoxide anion production [156], a key component for the phagocytic oxidative burst [157], which is a tool for destroying pathogens. In the context of NAFLD, a wide variety of cells increase superoxide production, including the aforementioned Kupffer cells, as well as neutrophils and other granulocytes [158], which may contribute to NAFLD-associated oxidative stress. Furthermore, NAFLD is associated with increased aerobic glycolysis [159], leading to increased lactate production and stabilization of HIF-1α (hypoxia-inducible factor 1α). This HIF-1α stabilization, in turn, promotes usage of the glycolytic pathway and promotes liver fibrosis in murine models of NAFLD [160], potentially signaling a key step in the transition from NASH to cirrhosis.

A further aspect of the immune system associated with NAFLD and progression to NASH is the NLRP3 (NOD-, LRR- and pyrin domain-containing protein 3) inflammasome. The NLRP3 inflammasome is an intracellular structure that senses microbial compounds and environmental stress. Its assembly leads to production of IL-1β and IL-18, as well as caspase-1-dependent apoptosis [161]. In mouse models of NASH, administration of a selective NLRP3 inhibitor suppressed caspase-1 and IL-1β accumulation, and limited the development of fibrosis [162], suggesting that the inflammasome plays a key role in mediating the progression of NAFLD. One of the major drivers of the inflammasome is ROS (reactive oxygen species) production, generated by OXPHOS. The mitochondrial electron transport chain (ETC) is an essential structure for ATP generation and is composed of multiple complexes (complexes I, II, III, IV and ATPase). Complex II, also known as succinate dehydrogenase, links the ETC with the TCA cycle, and chemical inhibition of this subunit prevents NLRP3 inflammasome activation [163]. Whilst there is evidence to suggest that ROS production leads to activation of the NLRP3 inflammasome [164], recent work suggests that it is ATP generation, and not ROS, that is required for inflammasome activation [163]. Since NAFLD and NASH are prevalent in patients with concurrent obesity and metabolic dysfunction, increased metabolic cycling through glycolysis and OXPHOS may exacerbate inflammasome activation and oxidative damage.

Understanding the immune response mechanism in NAFLD is crucial for developing a complete picture of sexual dimorphism in liver disease. As discussed previously in this review, there is evidence suggesting that androgens and estrogens both play a role in modulating the immune response, although many of the mechanisms by which they do this remain unclear or controversial. This gap in our knowledge of liver disease pathogenesis is one that needs filling urgently, as it has critical implications for the development of therapeutic strategies.

## 4. Current Non-Alcoholic Fatty Liver Disease In Vitro Modeling Systems

### 4.1. Overview of Non-Alcoholic Fatty Liver Disease in Humans

**Figure 6:**
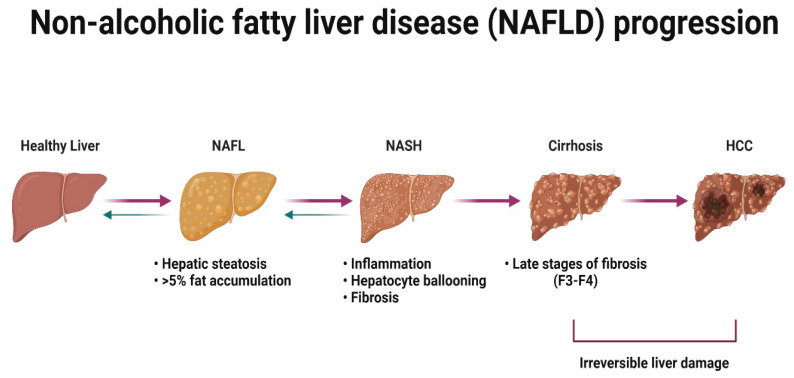
NAFLD progression. Schematic overview of NAFLD progression. Accumulation of >5% fat within the liver leads to hepatic steatosis (NAFL). In some patients, steatosis progresses to the inflammation and, consequently, the fibrosis stage, called NASH. NASH can further progress to the development of liver cirrhosis and eventually lead to liver cancer (HCC). NAFLD, non-alcoholic fatty liver disease; NAFL, non-alcoholic fatty liver; NASH, non-alcoholic steatohepatitis; F3–F4, fibrosis stages 3 and 4; HCC, hepatocellular carcinoma.

NAFLD is a condition defined by the excess accumulation of fat within the liver [165]. In the initial stages of the disease, there is little or no sign of inflammation or liver damage [166]. However, it is important to treat the benign stage of the disease because disease progresses in approximately one-third of patients [167]. NASH is associated with lobular and portal inflammation, hepatocyte ballooning and fibrosis [168,169,170], and can progress to cirrhosis [171] and hepatocellular carcinoma (HCC) in some patients [172] (Figure 6).

Recent meta-analysis and systematic review have concluded that the current overall prevalence of NAFLD worldwide is 32.4% [173], when 7 years ago it was ~24% [174]. The overall prevalence is significantly higher in men compared to women [173]. NASH has been identified in ~12% of the U.K. population [175] and was the second leading cause for liver transplantation in the U.S. in 2016 [176]. Younger patients with NASH seem to be predominantly men, however, after 50–60 years of age, the prevalence of NASH occurs more frequently in women [111,177]. Despite the increasing global burden, there are no licensed therapies for NAFLD or NASH [178,179]. There are several drug candidates for metabolic homeostasis currently in clinical trial phase III that are expected to end in 2025 (obeticholic acid) and in 2028 (semaglutidine), and several drugs against NASH in phase II [179]. Drug development in this space has been notoriously difficult and the phrase NAFLD/NASH graveyard has been coined. To improve success rates, the field needs to develop more physiologically relevant models, with a focus on human biology, diversity and the microbiome. Below, we review the advantages and disadvantages of current cell- and tissue-based systems pertinent to the study of human NAFLD/NASH ‘in the dish’.

### 4.2. Cell-Based Modeling of NAFLD

The hepatocyte is the key cell type involved in liver metabolism, with primary human hepatocyte (PHH) considered the gold standard for in vitro research on liver [180,181,182]. Although highly relevant, the availability of healthy human liver tissue is extremely limited and PHHs are mainly derived from transplant reject organs which could introduce artifacts into subsequent in vitro modeling studies [181,183]. Another limitation of working with PHHs is their instability in cell culture [180,181,184]. To overcome these limitations, multiple approaches have been employed, including various 3D culture configurations [185,186,187,188]. However, the models are still expensive to establish and relatively short-term in nature. To overcome the issues associated with PHHs, alternative primary cell sources have been the focus of other studies. Hepatic progenitors (HPCs) have been isolated from liver tissue. They are both expandable and are bipotent, resulting in differentiation to either cholangiocytes or hepatocytes [189,190]. Additionally, PHHs have been chemically induced in vitro to form expandable hepatic progenitor populations (CLiPs) [191,192,193]. The workhorse of the liver field are human cell lines established from transformed tissue and include HepG2, HepaRG and Huh7 [194]. The benefits of using cell lines are their affordable maintenance and expansion, and therefore they have been used extensively in NAFLD studies [195]. However, they do harbor numerous limitations which include incomplete phenotype, altered epigenetic patterns and karyotypic abnormalities.

### 4.3. Genetically Defined Models of Liver Steatosis

Pluripotent stem cells (PSCs) are a self-renewing stem cell population capable of differentiation into all human somatic cells [195]. The attraction of using this resource is that it is possible to capture sex and genetic diversity and the cell products are renewable. Since isolation of PHHs and their rapid de-differentiation in culture have limitations for research purposes, PSCs provide a suitable alternative [196]. Moreover, it is possible to create human tissue containing multiple cell types on the same genetic background. The field has bloomed over the last 20 years, with technologies becoming more affordable and reproducible. The simplest way to model NAFLD ‘in the dish’ is to use 2D PSC hepatocyte cultures and expose them to an excess of saturated and unsaturated free fatty acids (FFAs) and energy substrates. Such NAFLD model revealed transcriptomic alterations in genes involved in TCA cycle and OXPHOS, endoplasmic reticulum stress pathways and allowed to take a closer look at the benchmarks involved in NAFLD progression, such as de novo lipogenesis [197,198,199,200]. Despite their amenability to high-throughput screening, 2D hepatocyte cultures fail to replicate key cell to cell interactions between hepatocytes and non-parenchymal cells of the liver, resulting in altered hepatocellular structure, phenotype and disease modeling capacity. Therefore 3D models have been developed. Takebe et al. initially built liver tissue using a mix of PSC and primary cells, before using a liver tissue engineering product from only PSCs to accurately model hepatic steatosis [201,202,203]. Recently, a new 3D iPSC-derived liver organoid was developed which also contains stellate cells and Kupffer cells [203]. Treatment of these organoids with free fatty acids induced a steatohepatitis-like phenotype with concurrent development of fibrosis. These exciting developments are moving the field closer to being able to modulate immune–endocrine interactions, in a human-relevant setting, to identify new medicines to treat disease.

## 5. Concluding Remarks

Chronic metabolic dysfunction, especially obesity and T2D, is associated with the development of NAFLD. Clinical evidence from humans and studies in animal models have demonstrated clear evidence of sex bias in pathways affecting NAFLD establishment, progression and highlight the interplay between liver homeostasis, immune response and sex hormone signaling. Sex hormone correction of liver metabolism is the rationale for HRT. Although this has proven successful, improving liver function and reducing inflammation, the long-term use of HRT does come with increased health risks. Therefore, the identification of more targeted treatment strategies is required, necessitating the development of human models which accurately capture the interplay of metabolic syndrome, tissue fibrosis and the immune system.

## Data Availability

Not applicable.

## Abbreviations

17β-HSD	17β-hydroxysteroid dehydrogenase
3β-HSD	3β-hydroxysteroid dehydrogenase D5-D4 isomerase
*Acaca*	acetyl-CoA carboxylase alpha
ALT	alanine transaminase
AR	androgen receptor
ARE	androgen response element
CLiP	chemically induced hepatic progenitor
CXCL10	C-X-C motif ligand 10
CYP11A1	cytochrome P450 cholesterol side-chain cleavage enzyme
CYP17A1	cytochrome P450 17α-hydroxylase
CYP19A1	cytochrome P450 aromatase
CYP1A2	cytochrome P450 1A2
CYP3A4/5	cytochrome P450 3A4/5
CYP450	cytochrome P450 family
CYP4A1	cytochrome P450 4A1
DAMPs	damage-associated molecular patterns
DHEA	dehydroepiandrosterone
DHEA-S	dehydroepiandrosterone sulphate
DHT	dihydrotestosterone
E1	estrone
E2	estradiol
E3	estriol
E4	estetrol
ECM	extracellular matrix
EGF	epidermal growth factor
ERE	estrogen response element
ERα	estrogen receptor alpha
ERβ	estrogen receptor beta
ETC	electron transport chain
FFAs	free fatty acids
GLUT	glucose transporters
GPER1	G protein-coupled estrogen receptor 1
HCC	hepatocellular carcinoma
HDL	high-density lipoprotein
HIF-1α	hypoxia-inducible factor 1α
HPC	hepatic progenitor cell
HRE	hormone response element
HRT	hormone replacement therapy
HSC	hepatic stellate cell
IL	interleukin
LDL	low-density lipoprotein
LPS	lipopolysaccharide
MetS	metabolic syndrome
NAFLD	non-alcoholic fatty liver disease
NASH	non-alcoholic steatohepatitis
NLRP3	NOD-, LRR- and pyrin domain-containing protein 3
OXPHOS	oxidative phosphorylation
PHH	primary human hepatocyte
PPAR	peroxisome proliferator-activated receptor
PPP	pentose phosphate pathway
PSC	pluripotent stem cell
ROR	retinoid orphan receptor
ROS	reactive oxygen species
*Scd1*	stearoyl-CoA desaturase 1
SHBG	sex hormone-binding globulin
T	testosterone
T2D	type 2 diabetes
TCA	tricarboxylic acid
T_H_	T helper cell
TLR4	toll-like receptor 4
TNFα	tumour necrosis factor alpha
TNFR1	TNFα receptor
